# CRAFITY and PALBI Define a Machine Learning-Supported Prognostic Framework in Hepatocellular Carcinoma—Data from an Eastern European Cohort with Low Macrotrabecular-Massive Prevalence

**DOI:** 10.3390/diseases14070234

**Published:** 2026-06-29

**Authors:** Cristiana Grapa, Tudor Mocan, Daniel Leucuta, Rares Craciun, Lavinia-Patricia Mocan, Miroslaw T. Kornek, Emil Mois, Nadim Al Hajjar, Florin Graur, Teodora Mocan, Zeno Sparchez

**Affiliations:** 1 Department of Physiology, “Iuliu Haţieganu” University of Medicine and Pharmacy, 400012 Cluj-Napoca, Romania; grapa.cristiana.maria@elearn.umfcluj.ro (C.G.); teodora.mocan@elearn.umfcluj.ro (T.M.); 2 Gastroenterology Clinic, “Prof. Dr. O. Fodor” Regional Institute of Gastroenterology and Hepatology, 400162 Cluj-Napoca, Romania; tudor.mocan@ubbcluj.ro (T.M.); zsparchez@elearn.umfcluj.ro (Z.S.); 3 UBBmed Department, Babeș-Bolyai University, 400084 Cluj-Napoca, Romania; 4 Institute of Molecular Medicine and Experimental Immunology, University Hospital of the Rheinische Friedrich-Wilhelms-University, 53127 Bonn, Germany; miroslawkornek@web.de; 5 Department of Medical Informatics and Biostatistics, “Iuliu Haţieganu” University of Medicine and Pharmacy, Pasteur Street, No. 6, 400349 Cluj-Napoca, Romania; dleucuta@umfcluj.ro; 6 Department of Internal Medicine, “Iuliu Haţieganu” University of Medicine and Pharmacy, 400012 Cluj-Napoca, Romania; 7 Department of Histology, “Iuliu Haţieganu” University of Medicine and Pharmacy, 400349 Cluj-Napoca, Romania; trica.lavinia@umfcluj.ro; 8 Department of General, Visceral and Thoracic Surgery, German Armed ForcesCentral Hospital, 56072 Koblenz, Germany; 9 Department of Surgery, “Iuliu Haţieganu” University of Medicine and Pharmacy, 400162 Cluj-Napoca, Romania; drmoisemil@elearn.umfcluj.ro (E.M.); nadim.alhajjar@umfcluj.ro (N.A.H.); florin.graur@umfcluj.ro (F.G.); 10 Nanomedicine Department, Regional Institute of Gastroenterology and Hepatology, 400162 Cluj-Napoca, Romania

**Keywords:** hepatocellular carcinoma, macrotrabecular-massive, machine learning, non-invasive scores, alpha-fetoprotein, C-reactive protein, platelets, albumin, bilirubin

## Abstract

**Background and Aims**: To develop an inclusive, predictive framework for hepatocellular carcinoma patients, beyond what the Barcelona Clinic Liver Cancer (BCLC) staging system captures alone, non-invasive scores have emerged as potential contributors. Among them, the CRAFITY score (CRP and AFP in ImmunoTherapY), originally developed for immunotherapy-treated HCC populations, and the Platelet-Albumin-Bilirubin (PALBI) score have shown promising prognostic performance in selected cohorts. Likewise, the macrotrabecular-massive (MTM) histological subtype has been identified as a strong independent predictor of tumor recurrence, particularly in surgical series; whether it retains the prognostic significance in a mixed-treatment cohort remains unexplored. We aimed to evaluate the independent prognostic performance of CRAFITY and PALBI across all HCC treatment modalities, determine MTM prevalence and assess whether histological subtyping adds prognostic value beyond these readily available clinical scores. **Methods**: The study included 500 consecutive, pathologically confirmed HCC patients at a tertiary gastroenterology center in Cluj-Napoca, Romania. MTM subtype was defined as >50% macrotrabecular architectural pattern on histological review by two senior pathologists. Overall survival (OS) and recurrence-free survival (RFS) were assessed by Kaplan–Meier analysis and multivariable Cox regression. A random survival forest (RSF) model was constructed to identify dominant prognostic predictors. **Results**: MTM was identified in 14 patients (2.8%) and did not independently predict OS (HR 0.94, 95% CI 0.49–1.81, *p* = 0.85) or recurrence (OR 3.78, *p* = 0.116). In this heterogeneous cohort spanning multiple treatment modalities, CRAFITY (HR 1.68, 95% CI 1.42–1.98, *p* < 0.001) and PALBI (HR 1.51, 95% CI 1.22–1.87, *p* < 0.001) were strong independent predictors of OS after BCLC stage. RSF analysis confirmed this hierarchy with a C-index of 0.734. **Conclusions**: CRAFITY and PALBI demonstrated strong, independent predictive performance for a large, underrepresented, heterogenous Eastern European HCC cohort. In contrast, MTM subtype showed limited prognostic value in this cohort. The results support the broader applicability of CRAFITY beyond its original immunotherapy context and underline the low prevalence of MTM subtype.

## 1. Introduction

Hepatocellular carcinoma is one of the leading causes of cancer-related deaths worldwide, accounting for more than 80% of liver cancers, representing an increasing global health burden, despite extensive and promising studies [1]. As HCC etiologies shift from viral to metabolic and alcohol-related liver disease, resulting in an increase in biological heterogeneity, this transition can challenge current risk stratification models [2].

Therapeutic decision management in HCC is guided by the Barcelona Clinic Liver Cancer (BCLC) staging system [3]; their architecture has been repeatedly validated and adopted by major guidelines, representing the pillar from which therapy selection and survival prediction starts for the HCC patient. While this staging system still represents the cornerstone for clinical decision making, it primarily relies on liver function, tumor burden and performance status, and it does not fully capture the biological heterogeneity of HCC [4]. This limitation is reflected by the variability in outcomes that is encountered at an individual level. Given the multifactorial nature of treatment decisions, one significant challenge is to comprehensively include all significant determinants to patient prognosis.

Among the non-invasive tools that could improve prognostication for HCC and challenge this limitation, liver function-based scores have attracted considerable attention. The albumin-bilirubin (ALBI) and its derivative PALBI emerged, showing promising prognostic value across the spectrum of HCC patients [5,6,7]. Beyond liver function-based scores, another easily applicable score, namely CRAFITY score, was initially developed as a prognostic tool for HCC patients receiving immunotherapy [8] and was also validated in other subgroups as a potentially valuable scoring system for informed clinical decisions [9]. The score comprises the following two complementary biomarkers: C-reactive protein (CRP) and alpha-fetoprotein (AFP), their combination capturing two distinct biological dimensions that are not represented in the BCLC staging system. To our knowledge, the CRAFITY score has never been evaluated in a mixed-treatment cohort that encompasses the full spectrum of HCC management; whether its prognostic utility is limited to the immunotherapy context for which it was developed, or it captures a more expanded dimension, is a clinically important question yet to be addressed.

In parallel, the 2019 5th edition of the World Health Organization (WHO) Classification of Tumors of the Digestive system [10] has recognized distinct histological HCC subtypes that together comprise about 35% of HCCs and include the macrotrabecular massive subtype. This particular subtype has since been associated with a poor prognosis (higher tumor burden, presence of vascular invasion and satellite nodules, and higher AFP levels) [11,12] and high recurrence risk [13]. However, within a unified frame and outside selected cohorts, it is still unclear whether histologic subtyping adds real value to prognostication of HCC patients.

Given the limitations of the current staging systems, our study aimed to investigate whether non-invasive scores such as CRAFITIY and PALBI can outperform histological subtyping in a heterogenous HCC cohort. We hypothesize that in a real-world HCC population, the prognostic signal attributed to MTM morphology would be diminished when compared to clinical scores that could relatively capture the same biological dimensions. The rationale behind the hypothesis relies on the potential biological convergence with the molecular features of MTM subtype: MTM-HCC has been associated with TP53 mutations and FGF19 amplification, which can lead to elevated AFP levels [12,13], whereas CRAFITY incorporates AFP levels and can directly capture this feature. Additionally, the systemic inflammation reflected by the CRP component of CRAFITY can be linked to the highly vascular MTM phenotype [13]. Regarding PALBI, its surrogate role for portal hypertension is relevant since MTM subtype arises predominantly in a non-cirrhotic background [12]. Therefore, both approaches may partially reflect an overlap of the tumor aggressiveness, from different perspectives. The study is conducted on a series of 500 pathologically confirmed HCC patients, from a geographically underrepresented population in the HCC literature, during a ten-year period that marks the epidemiological shift from viral to non-viral HCC etiology.

## 2. Materials and Methods

### 2.1. Study Design and Setting

This retrospective study included 500 patients with hepatocellular carcinoma, diagnosed either through liver biopsy or liver resection, between 2015 and 2025, at the Regional Institute of Gastroenterology and Hepatology, “Prof. Dr. Octavian Fodor” in Cluj-Napoca, Romania. The study was conducted in accordance with the Declaration of Helsinki and approved by the Institutional Ethics Committee. Informed consent was waived given the retrospective nature of the study and the anonymization of patient data.

### 2.2. Participants

We retrospectively included a mixed, single-center, consecutive cohort of 500 patients who were diagnosed with HCC in various stages (from BCLC 0 to BCLC D), pathologically confirmed either through liver biopsy or liver resection, at a tertiary center in Cluj-Napoca, Romania, during a 10-year period (2015–2025).

Exclusion criteria were represented by incomplete (R1 or R2) resection for patients who underwent liver resection, unavailable or inadequate histological slides (necrosis > 90%), histological features indicative for combined hepatocellular-cholangiocellular carcinoma, preoperative antitumoral treatment or prior treatment to first diagnosis in our clinic. Patients with incomplete (R1 or R2) resections were excluded due to the fact that residual disease is a major determinant for disease recurrence and survival, independent of tumor biology; likewise, patients with prior treatments would present with a potentially different tumor morphology, altered by the effects of the treatment received—their exclusion ensures a baseline, homogenous evaluation of the patient cohort.

### 2.3. Data Sources and Measurement

Every histological slide was reviewed by two senior pathologists, blinded to clinical outcomes. Macrotrabecular subtype was defined as a tumor with predominantly (>50%) macrotrabecular (trabeculae of more than six cells thick) architectural pattern. Interobserver agreement for MTM classification was assessed using Cohen’s unweighted kappa coefficient. Discordant cases were resolved by consensus review at a joint session.

Patients treated with surgical resection or microwave/radiofrequency ablation (MWA/RFA) were monitored with periodic imaging. The clinical endpoints were early, overall tumor recurrence and overall survival. Early recurrence was defined as the appearance of one or more enhancing lesions on imaging within 24 months following resection or ablation.

### 2.4. Variables

Exploratory variables were noninvasive scores such as CRAFITY and PALBI, and MTM histological subtype. As prespecified confounders we used age at diagnosis (years), BCLC stage, Child–Pugh score, MELD score, Edmondson–Steiner tumor grade, esophageal varices, splenomegaly, RFA/MWA, TACE, and sorafenib treatment.

The CRAFITY score was calculated using CRP and AFP levels, with each parameter contributing 0 or 1 points (total range 0–2), with one point assigned for CRP ≥ 1 mg/dL and one point assigned for AFP ≥ 100 ng/mL, and categorized as 0, 1 or 2. The PALBI score was calculated using the following formula: (2.02 × log_10_ bilirubin) + [−0.37 × (log_10_ bilirubin)^2^] + (−0.04 × albumin) + (−3.48 × log_10_ platelets) + [1.01 × (log_10_ platelets)^2^] and classified into the following three grades: grade 1 (≤−2.53), grade 2 (−2.53 to −2.09), and grade 3 (>−2.09).

### 2.5. Statistical Analysis

All analyses were conducted in R (version 4.3.2). Continuous variables were expressed as median (IQR) and categorical variables as counts and percentages. Group comparisons were performed using the Mann–Whitney U or Kruskal–Wallis test for continuous variables and the chi-square or Fisher’s exact test for categorical variables.

Overall survival and recurrence-free survival (RFS) were estimated using the Kaplan–Meier method and compared with the log-rank test. Cox proportional hazards regression was used for univariate and multivariate analyses, with results reported as hazard ratios (HRs) and 95% confidence intervals (CIs). For the recurrence analysis, data were available for 217 out of 500 patients who underwent curative treatment and had at least one follow-up imaging study confirming complete response; this sub-cohort was predominantly BCLC 0-A (97.9% of patients without recurrence, 91.7% of patients with recurrence). Regarding the missing data, patients either received non-curative treatment where the concept of recurrence does not apply, or they lacked adequate follow-up. Patients who received more than one treatment modality (e.g., resection followed by ablation for a recurrent lesion, or combined resection and RFA) were included and counted under each applicable treatment category. The prognostic impact of the MTM subtype was assessed in both univariate and multivariate models. For the recurrence analysis, logistic regression was applied to evaluate factors associated with recurrence occurrence. Recurrence-free survival was evaluated separately using Kaplan–Meier methods. Given the limited sample size of the recurrence cohort and the small number of MTM cases with available recurrence data, a multivariable Cox model for RFS was not applied due to concerns regarding model stability and overfitting.

Survival outcomes were modeled using a random survival forest method from randomForestSRC package in R. The analysis included 13 prespecified independent variables, which included possible biomarkers that could predict survival, as well as the following known variables that influence survival: age at diagnosis (years), PALBI score, CRAFITY score, BCLC stage, Child–Pugh score, MELD score, Edmondson–Steiner tumor grade, MTM histological subtype, esophageal varices, splenomegaly, RFA/MWA, TACE, and sorafenib treatment. Missing data were handled internally using adaptive tree imputation, which iteratively imputes missing values based on proximity within the forest. Missingness among the RSF model variables was limited as follows: CRP values were missing for 20 patients (4.0%), AFP in 12 patients (2.4%), and Edmondson–Steiner grade in one patient (0.2%), with all remaining variables complete. A complete-case sensitivity analysis was not performed, as only 26 patients (5.2%) had missing data in any RSF covariate, resulting in a complete-case dataset comprising 94.8% of the study population. Hyperparameter tuning was performed over a predefined grid to optimize model performance. The following values were evaluated: number of trees (ntree) = 500 and 1000; number of variables randomly selected at each split (mtry) = 3, 4, and 5; and minimum terminal node size (nodesize) = 10, 15, and 20. Model selection was based on out-of-bag (OOB) prediction performance, with the optimal combination of hyperparameters retained for the final model. The final RSF model was refit using the selected optimal parameters and included variable importance estimation based on permutation importance measures. Model performance was evaluated using Harrell’s concordance index (C-index), computed from out-of-bag (OOB) predictions.

Statistical significance was set to 0.05, for two tailed *p* values.

## 3. Results

A total of 500 patients with hepatocellular carcinoma were included in the analysis. The median age at diagnosis was 65 years (IQR 60–71). The macrotrabecular-massive subtype was identified in 14 patients (2.8%), with an interobserver agreement of κ = 0.75.

### 3.1. Comparison Between MTM and Non-MTM Subtypes

Baseline characteristics are summarized in Table 1. MTM tumors were more frequently diagnosed by biopsy (78.6% vs. 47.9%, *p* = 0.024). They were associated with cryptogenic etiology (28.6% vs. 8.9%, *p* = 0.034) while hepatitis C infection was less frequent (7.1% vs. 38.5%, *p* = 0.017).

MTM patients had a lower prevalence of splenomegaly (21.4% vs. 59.1%, *p* = 0.005) and a lower borderline rate of cirrhosis (50% vs. 76.3%, *p* = 0.051). No significant differences were observed regarding BCLC stage, tumor characteristics, or treatment allocation. A higher proportion of MTM tumors were associated with elevated AFP levels (≥100 ng/mL) compared to non-MTM cases (53.8% vs. 31.4%); however, this difference did not reach statistical significance (*p* = 0.128). In contrast, no differences were observed in tumor stage distribution, with similar proportions of early-stage disease according to the BCLC staging system (71.4% vs. 64.8%, *p* = 0.779). Additional laboratory characteristics are presented in Supplementary File Table S1. Kaplan–Meier analyses showed no significant differences in recurrence-free survival (Supplementary File Figure S1) or overall survival (Supplementary File Figure S2) between MTM and non-MTM groups. Early recurrence rates were also similar (12.5% vs. 35.9%, *p* = 0.266).

### 3.2. Recurrence Subgroup Analysis

Among 217 patients with available data, recurrence occurred in 120 patients (55.3%). Recurrence was significantly associated with the presence of esophageal varices (*p* = 0.003), splenomegaly (*p* = 0.003), and BCLC stage (*p* = 0.037), with higher ALBI values (median −2.57 vs. −2.67, *p* = 0.023). Treatment modalities differed significantly, with higher rates of RFA/MWA, TACE, sorafenib, and immunotherapy in patients with recurrence (all *p* < 0.05). MTM status was not associated with recurrence (*p* = 0.734) (Table 2).

In multivariate logistic regression (Table 3), splenomegaly (adjusted OR 2.76, 95% CI 1.17–6.74, *p* = 0.022) was an independent predictor of recurrence. MTM subtype was not independently associated with recurrence (*p* = 0.116). The model demonstrated good calibration, with no evidence of lack of fit (Hosmer–Lemeshow test *p* = 0.979). The overall correct classification rate was 71.89%.

### 3.3. Machine Learning-Based Survival Analysis

A random survival forest (RSF) model was constructed using 500 patients, including 351 deaths. The final model was built using 1000 trees, with mtry = 3 and node size = 15. The model demonstrated good discriminative ability, with an out-of-bag Harrell’s concordance index (C-index) of 0.734.

**Figure 1:**
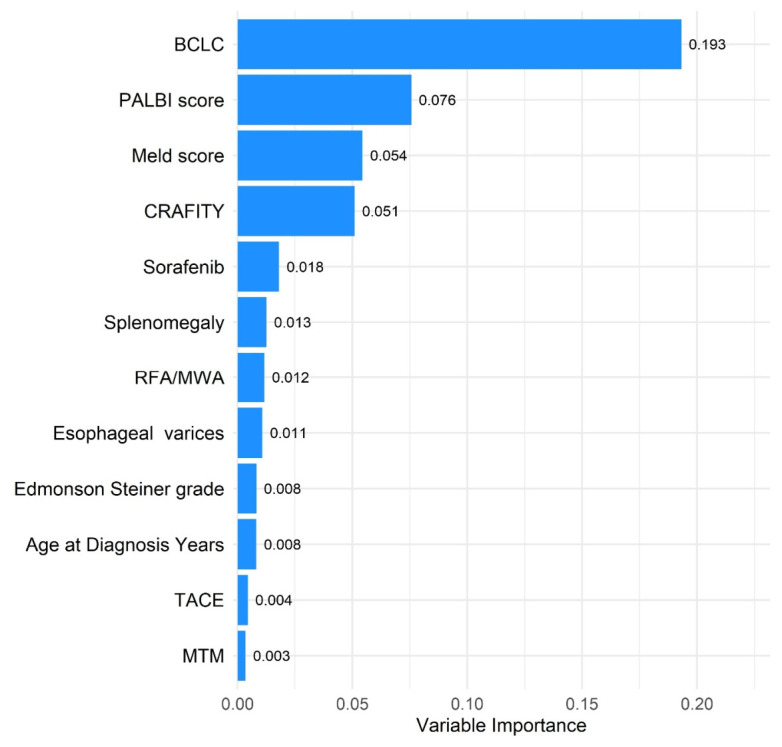
Variable importance plot from the random survival forest model for overall survival. Permutation-based variable importance analysis identified BCLC stage as the strongest predictor of overall survival, followed by PALBI, MELD, CRAFITY scores. The MTM subtype demonstrated limited contribution to model performance.

Variable importance analysis identified BCLC stage as the strongest predictor of survival, followed by liver function-related indices, including PALBI, MELD, and CRAFITY scores. Treatment-related and histopathological variables showed limited contribution to model performance (Figure 1).

**Figure 2:**
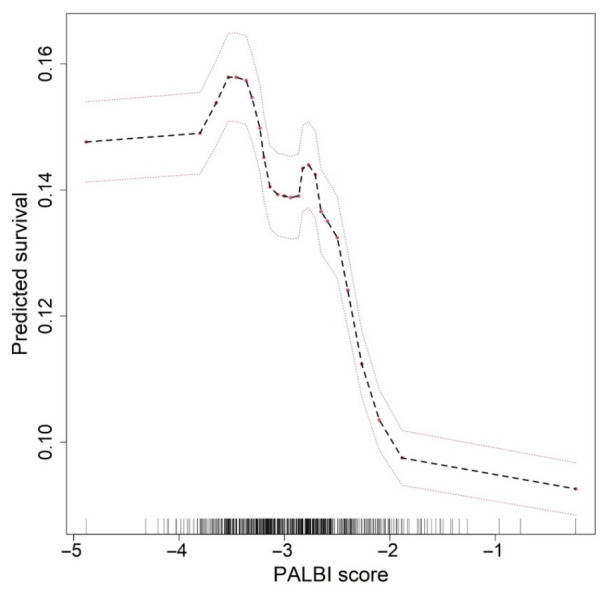
Partial dependence plot of PALBI score from the random survival forest model for overall survival. Increasing PALBI values were associated with progressively lower predicted survival probabilities, with evidence of nonlinear and threshold effects at higher score values.

**Figure 3:**
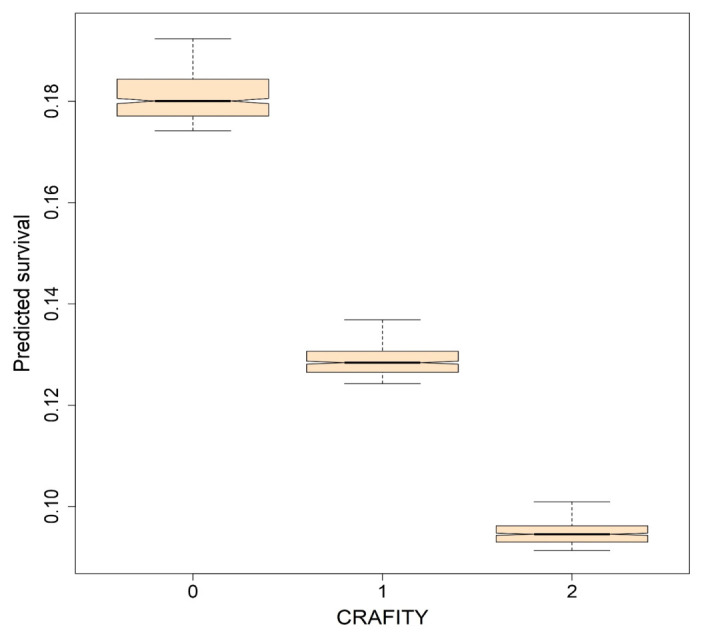
Partial dependence plot of CRAFITY score from the random survival forest model for overall survival. Higher CRAFITY categories were associated with progressively lower predicted survival probabilities, demonstrating a graded relationship between systemic inflammation/tumor biomarker burden and survival outcome.

Partial dependence analysis demonstrated nonlinear associations between liver function indices and predicted survival probabilities. Increasing PALBI values was associated with progressively lower predicted survival, with evidence of threshold effects (Figure 2). CRAFITY score showed a graded relationship with survival, with decreasing predicted survival across increasing categories (Figure 3).

**Figure 4:**
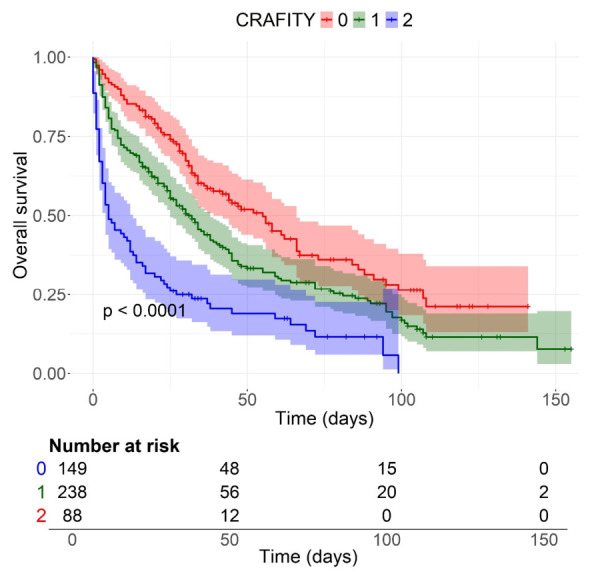
Kaplan–Meier overall survival curves according to CRAFITY score. Overall survival differed significantly across CRAFITY categories, with progressively lower survival observed in patients with higher scores.

**Figure 5:**
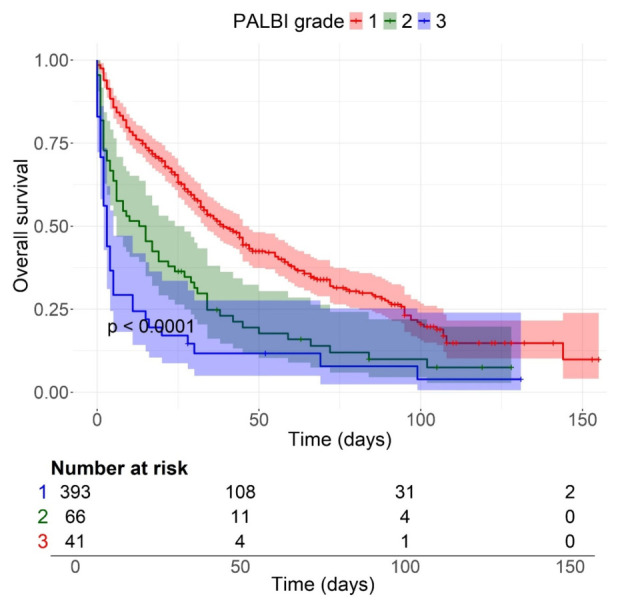
Kaplan–Meier overall survival curves according to PALBI grade. Higher PALBI grades were associated with significantly reduced overall survival compared with lower PALBI grades. Survival probabilities were estimated using the Kaplan–Meier method and compared using the log-rank test.

A similar nonlinear association was observed for MELD score, with decreasing predicted survival at higher values (Supplementary File Figure S3). BCLC stage demonstrated a monotonic association with predicted survival, with progressively lower predicted survival probabilities from early (0/A) to advanced stages (C/D) (Supplementary File Figure S4). Kaplan–Meier survival analysis demonstrated differences in overall survival according to CRAFITY score, with progressively lower survival observed across increasing score categories (Figure 4). Similarly, overall survival differed according to PALBI grade, with reduced survival observed in higher PALBI grades (Figure 5).

### 3.4. Multivariable Survival Analysis

A multivariable Cox proportional hazard model was constructed to check if PALBI score, CRAFITY and MTM are independent predictors of overall survival, after adjustment for known predictors (age ≥ 65 years, BCLC group (late vs. early), Edmondson–Steiner grade II/III, esophageal varices, and splenomegaly) (Table 4). Age ≥ 65 years (HR 1.27, 95% CI 1.01–1.58, *p* = 0.039), advanced BCLC stage (HR 2.89, 95% CI 2.20–3.79, *p* < 0.001), PALBI score (HR 1.51, 95% CI 1.22–1.87, *p* < 0.001), and CRAFITY score (HR 1.68, 95% CI 1.42–1.98, *p* < 0.001) were independently associated with overall survival. Edmondson–Steiner grade II–III (HR 1.35, 95% CI 1.08–1.68, *p* = 0.009) and splenomegaly (HR 1.35, 95% CI 1.05–1.74, *p* = 0.021) were also independently associated with increased mortality risk. Esophageal varices showed a borderline association with survival (HR 1.28, 95% CI 0.99–1.66, *p* = 0.063). Treatment-related variables, including RFA/MWA (*p* = 0.91), TACE (*p* = 0.099), and sorafenib treatment (*p* = 0.536), were not independently associated with overall survival. MTM subtype was not associated with overall survival (HR 0.94, 95% CI 0.49–1.81, *p* = 0.85).

## 4. Discussion

The macrotrabecular-massive HCC subtype was identified in 14 patients (2.8%). This prevalence is substantially lower than the 12% (16% of surgically resected samples, 8.5% of liver biopsy samples) reported in the study that was the first formal clinical validation for MTM [12]. The study confirmed MTM as an independent predictor of early recurrence (HR 3.03) and overall recurrence (HR 2.76) in multivariate analysis, and associated MTM with elevated AFP levels, larger tumor size, the presence of satellite nodules and vascular invasion. The wide range of prevalence reports from various studies (8.5–38%) is informative, as it may reflect a combination of case selection, underlying etiology and diagnostic method. To expand, a recent multicenter contrast enhanced ultrasound (CEUS) study cited rates of 10.0–38.2%, with higher proportions in regions with an elevated HBV prevalence [14], while in Western and mixed-etiology surgical cohorts, reported prevalence varied from 7.1% to 9.2% [15,16,17]. Thus, the 2.8% prevalence observed in our cohort is consistent with the sampling effect, given that 78.6% of our MTM diagnoses (versus 47.9% of non-MTM cases (*p* = 0.024)) was established on biopsy material. This methodological consideration should be acknowledged, as selected surgical cohorts could potentially overestimate MTM prevalence at a population level [18].

The molecular landscape of MTM is characterized by tumor protein53 (TP53) alterations and Fibroblast Growth Factor 19 (FGF19) amplification, which are suggestive for non-viral, metabolic backgrounds [11]; this is consistent with our findings regarding etiology—in our MTM group, cryptogenic disease was predominant (28.6% vs. 8.9%, *p* = 0.034) with a low HCV infection (7.1% vs. 38.5%, *p* = 0.017). Additionally, splenomegaly as a marker for portal hypertension was significantly less frequent in the MTM group (21.4% vs. 59.1%, *p* = 0.005) and cirrhosis showed a borderline difference (50% vs. 76.3%, *p* = 0.051), in harmony with the known tendency of metabolic HCC to arise on a less fibrotic background [19]. These findings are of value as the shift from viral to non-viral etiology continues at a global level, and the molecular substratum conducive to MTM-like morphology may in fact become more prevalent.

On Kaplan–Meier analysis, neither overall survival nor recurrence-free survival differed significantly between MTM and non-MTM patients; in multivariate models, MTM status also failed to emerge as an independent predictor of either recurrence or overall survival. This contradicts current literature reports that indicate a strong independent predictive value for both early and overall recurrence [12,13,18]. The divergence from this narrative could be explained by the main limitation of our study—the small number of MTM cases (n = 14) which renders limited statistical power for time to event analysis. Only 8 of the 14 MTM patients in our cohort had complete recurrence follow-up data, so the recurrence analysis was an exploratory endpoint that was insufficiently powered for definitive inference; results should be interpreted as hypothesis-generating only. Additionally, the absence of an independent association between MTM status and clinical outcomes should not be interpreted as evidence of a lack of prognostic relevance. Rather, the results should be viewed in the context of the very low prevalence of MTM cases in our cohort and the limited statistical power available for MTM-specific analyses.

The numerically higher proportion of AFP ≥ 100 ng/mL in MTM patients (53.8% vs. 31.4%, *p* = 0.128) is clinically consistent with the expected aggressive biology, remaining non-significant also due to the small sample size. Additionally, the heterogeneity of our cohort that included patients in all stages who underwent various types of curative and palliative treatments inevitably introduced prognostic variability that dilutes the contribution of morphological features. With only 14 MTM patients in our cohort, the study was critically underpowered, achieving approximately 15% power for overall survival analysis; adequate power (80%) would have required approximately 80 MTM patients, a prevalence of 16% that is consistent only with purely surgical series. We therefore postulate that the prognostic relevance of MTM subtype is context-dependent, with a marked attenuation of its role in non-surgical cohorts.

Regarding the recurrence analysis, among the 217 patients with available data, splenomegaly (adjusted OR 2.76, 95% CI 1.17–6.74, *p* = 0.022) emerged as the only independent predictor of tumor recurrence in multivariate logistic regression, while the presence of esophageal varices was significantly associated with recurrence on univariate analysis (*p* = 0.003). These findings are consistent with the well-established prognostic role of portal hypertension in HCC: the presence of esophageal varices has been shown to independently predict poorer survival in large multicenter series [20,21], while splenic volume was identified as an independent predictor of late recurrence after hepatectomy in HCC patients [22]. The strong association between ablative therapies and recurrence in univariate analysis most reasonably reflects a surveillance-length bias, seeing as ablative therapies achieve complete local response in early-stage disease and thereby permit longer follow-up periods in which intrahepatic recurrence can be detected; this an interpretation consistent with published data showing that portal hypertension, rather than the therapeutic modality itself, is the primary recurrence driver in patients with HCC who underwent radiofrequency ablation [23]. The conjunction of our findings therefore reinforces a robust paradigm as follows: the hepatic microenvironment, which here was quantified through non-invasive surrogates such as splenomegaly and variceal status, is a central determinant of HCC outcome, independent of the intrinsic molecular subtype of the tumor.

In the Cox multivariate model analysis, the CRAFITY score emerged as one of the strongest independent predictors of overall survival with the Kaplan–Meier curve confirming progressively lower survival across increasing CRAFITY categories. The results represent an important external validation of the score in an Eastern European cohort encompassing multiple treatment modalities—a substantially broader clinical context than any prior validation study and, to our knowledge, the first of its kind. Originally developed for patients with HCC receiving anti-PD-(L)1–based immunotherapy [8], the score was then validated through a meta-analysis of 786 patients that confirmed that lower CRAFITY score independently predicted better OS (HR 0.22, 95% CI 0.10–0.50) and progression-free survival (HR 0.36, 95% CI 0.23–0.55) [24]. In locoregional settings, CRAFITY was tested in a cohort of patients who received combined TACE and immunotherapy [25], the study showing that a lower CRAFITY score was an independent predictor of both better overall survival (*p* = 0.045) and progression-free survival (*p* < 0.001) on multivariate analysis, with CRAFITY-low patients also achieving superior early tumor response at three months; their finding confirmed the prognostic utility of the score beyond its original immunotherapy context.

The components of CRAFITY score, namely CRP and AFP, are markers with widely accepted roles for HCC progression: while AFP encapsulates tumor burden and its secretory activity, with a validated prognostic role [26], CRP is indicative for the systemic inflammatory microenvironment that promotes tumor proliferation and angiogenesis [25]. A study on 2770 hepatectomy patients independently validated the superiority of their combination for OS and RFS prediction [27]. The study therefore provides a strong biological plausibility for CRAFITY application even in surgical cohorts. Of particular interest in the context of our study is the relationship between CRAFITY and the MTM phenotype. The trend we observed toward higher AFP ≥ 100 ng/mL in MTM patients (53.8% vs. 31.4%, *p* = 0.128) is directionally consistent with the established literature: elevated AFP is a characteristic feature of MTM-HCC in both Ziol et al. [12] and Calderaro et al. [11] datasets. At the molecular level, this phenomenon is explained through a pro-tumorigenic state that drives both an elevation in AFP production and a macrotrabecular morphology as follows: the genomic hallmarks of MTM-HCC are represent by TP53 mutations and FGF19 amplification which are associated with tumor proliferation and angiogenesis; these tumors frequently exhibit high AFP levels and a highly vascular phenotype, with overexpression of angiogenic factors such as VEGFA and ANGPT2, which may contribute to their aggressive clinical behavior [18]. This may indicate that CRAFITY’s AFP component could capture the same biological aggressiveness as MTM morphology. This convergence raises the following clinically important hypothesis: in patients where biopsy material is insufficient or when biopsy is not available, a high CRAFITY may potentially serve as a practical non-invasive signal of aggressive tumor biology consistent with, though not diagnostic of, the MTM phenotype, and may partly account for why MTM status loses independent prognostic significance when CRAFITY is included in a multivariate model. Given the limited number of MTM cases, this observation should be considered hypothesis-generating and requires validation in larger prospective cohorts.

The PALBI score was also independently associated with overall survival in our Cox multivariate model (adjusted HR 1.51, 95% CI 1.22–1.87, *p* < 0.001)—second only to BCLC stage among all variables tested—and Kaplan–Meier analysis demonstrated significantly reduced survival across progressively higher PALBI grades. As an addition to ALBI, the PALBI score integrates albumin, bilirubin, and platelet count, with the latter serving as an accessible surrogate of portal hypertension; it was designed explicitly to overcome known limitations of the Child–Pugh score, particularly its subjective components. In a nationwide Korean cohort study, PALBI achieved a larger AUROC for predicting OS than Child–Pugh, MELD, and ALBI scores (0.675 vs. 0.633, 0.645, and 0.642 respectively, *p* < 0.001) [28], and in a comparative analysis of albumin-based models in TACE patients, PALBI outperformed competing scores across all modalities [29]. A 2022 meta-analysis of 13 retrospective studies including 15,534 patients confirmed that high PALBI grade was significantly associated with poor OS (pooled HR 1.71, 95% CI 1.46–2.02) and poor disease-free survival (HR 1.31, 95% CI 1.11–1.54), with subgroup analyses confirming these findings across treatment modalities [30].

The independent contributions of PALBI and CRAFITY in our multivariate model illustrate the following canonical principle: HCC prognosis is mediated by an interplay between liver functional reserve (captured by PALBI), systemic inflammation (captured by CRAFITY), disease extent (BCLC stage), and host factors (age, splenomegaly, and Edmondson–Steiner grade). Within this multidimensional framework, MTM histological status adds no independent prognostic signal, underscoring the superior resolution offered by clinical and biochemical scoring over isolated pathological classification in mixed HCC cohorts. The reasoning behind the choice of specifically these two scores is hypothesis-driven, based on their ability to capture prognostic dimensions that are plausible surrogates for the tumor characteristics of MTM-HCC: including CRAFITY and PALBI in the same multivariate model is a potential test of whether MTM status could carry additional prognostic substrate beyond what these non-invasive scores already capture.

A random survival forest model was applied to the full cohort, to complement the Cox regression model, reaching an out-of-bag Harrell’s C-index of 0.734, a performance that is unconstrained, data-driven and stable in the presence of correlated predictors. More importantly, our results are consistent with literature reports on machine learning models for HCC prognosis (C-index 0.69–0.731) [31,32,33]. The analysis revealed BCLC stage is the dominant predictor of overall survival, followed by clinical and biochemical scores (PALBI, MELD, and CRAFITY), while MTM histological subtype showed negligible permutation variable importance. Regarding the contribution of the MELD score to OS prediction in the model, it was not specifically explored in additional survival analyses because the choice for its incorporation in the model was dictated by its role for evaluation of liver function severity, thus ensuring an adequate adjustment for it; additionally, due to the fact that the score’s variables overlap with PALBI, which was previously established as being superior to MELD in HCC cohorts [28], the prognostic signal attributed to MELD was not considered valuable in this context.

Overall, the study carries important strengths that align our findings within the current body of literature. First, this is one of the few analyses on the prevalence and prognostic impact of MTM subtype in an Eastern European, large, mixed-treatment population of consecutive patients, during a ten-year period—that being a reflection for contemporary clinical practice rather than carefully curated surgical series, which may be unrepresentative. Second, the multilayered statistical approach that combined Kaplan–Meier estimation, multivariate logistic regression, multivariate Cox regression and a machine learning RSF model allows our findings to be replicated, particularly regarding the noninvasive prognostic scores. Finally, to our knowledge, this is the first external validation of the CRAFITY score beyond its primary purpose, applied to an unselected cohort that is both geographically underrepresented in the existing literature and clinically heterogeneous, strengthening the case for its routine application across all HCC management contexts.

The limitations of our study also need to be acknowledged and accounted. The main limitation is represented by the small number of MTM cases, which hinders statistical power, leading to results that cannot be attributed to a true negative effect, due to insufficient sample size. The MTM recurrence analysis was underpowered for any definitive conclusion and should be considered exploratory and hypothesis-generating. In a retrospective, non-randomized dataset, where patients receive different types of treatment, multivariate analysis cannot completely account for residual confounding, while the mere retrospective nature leads to inherent risks of selection bias. The exclusion of patients with incomplete (R1 or R2) resection and prior treatments, while methodologically justified, led to a reduction in the representativeness of the cohort therefore influencing the generalizability of our results. Our selected cohort may likely present with a less aggressive tumor biology and better liver function leading to an overestimation of the prognostic gradient of the calculated scores. Additionally, despite our RSF model showing a good discrimination value, external validation is required before clinical application.

## 5. Conclusions

Our findings suggest routine, inexpensive, laboratory parameters can provide reproducible and reliable prognostic stratification, while histological subtypes may reflect biological processes that could be accurately captured through circulating biomarkers. The biological overlap between the two CRAFITY inputs and MTM morphology further raises the hypothesis that this score may serve as a noninvasive surrogate for an aggressive tumor type, a relationship which warrants prospective validation. While MTM was not independently associated with recurrence or survival in this cohort, the low number of MTM cases precludes definitive conclusions regarding its prognostic value and warrants confirmation in larger real-world cohorts. Prospective investigations are also needed to determine whether the prognostic value of CRAFITY extends consistently across treatment modalities and healthcare settings. Finally, future prognostic models may benefit from integrating histological, clinical, and machine learning-derived variables into unified risk stratification frameworks.

## Figures and Tables

**Table 1:** Comparison between MTM and non-MTM patients.

MTM-HCC Subtype:	Yes (*n* = 14)	No (*n* = 486)	*p*
Tissue sampling (biopsy), n (%)	11 (78.57)	233 (47.94)	0.024
Sex (female), n (%)	3 (21.43)	122 (25.1)	1
Recurrence (early), n (%)	1/8 (12.5)	75/209 (35.89)	0.266
NASH, n (%)	3 (21.43)	87 (17.9)	0.725
Cryptogen, n (%)	4 (28.57)	43 (8.85)	0.034
HCV, n (%)	1 (7.14)	187 (38.48)	0.017
HBV, n (%)	4 (28.57)	109 (22.43)	0.53
Alcohol, n (%)	2 (14.29)	116 (23.87)	0.536
AIH, n (%)	0 (0)	3 (0.62)	1
Other etiologies, n (%)	7 (50)	96 (19.75)	0.012
Cirrhosis, n (%)	7 (50)	371 (76.34)	0.051
Child–Pugh score, n (%)			0.072
A	7 (50)	320 (65.84)	
B	0 (0)	53 (10.91)	
No cirrhosis	7 (50)	113 (23.25)	
Esophageal varices, n (%)			0.225
No	13 (92.86)	348 (71.6)	
with bleeding	0 (0)	13 (2.67)	
Yes	1 (7.14)	125 (25.72)	
Ascites, n (%)	0 (0)	48 (9.88)	0.381
Splenomegaly, n (%)	3 (21.43)	287 (59.05)	0.005
BCLC, n (%)			0.364
0	4 (28.57)	70 (14.4)	
A	6 (42.86)	245 (50.41)	
B	2 (14.29)	72 (14.81)	
C	1 (7.14)	84 (17.28)	
D	1 (7.14)	15 (3.09)	
BCLC group (early), nr (%)	10 (71.43)	315 (64.81)	0.779
Portal vein invasion, n (%)	3 (21.34)	112 (23.05)	0.705
Satellite nodules, n (%)	4 (28.57)	25 (5.14)	0.204
Microvascular invasion, n (%)	8 (57.14)	94 (19.34)	1
Edmondson–Steiner grade, n (%)			0.191
	0 (0)	1 (0.21)	
I	0 (0)	38 (7.82)	
I/II	1 (7.14)	40 (8.23)	
II	4 (28.57)	174 (35.8)	
II/III	2 (14.29)	119 (24.49)	
III	5 (35.71)	99 (20.37)	
III/IV	2 (14.29)	12 (2.47)	
IV	0 (0)	3 (0.62)	
Liver resection, n (%)	10 (71.43)	236 (48.56)	0.092
LT, n (%)	0 (0)	2 (0.41)	1
RFA/MWA, n (%)	3 (21.43)	126 (25.93)	1
TACE, n (%)	1 (7.14)	30 (6.17)	0.597
SIRT, n (%)	0 (0)	2 (0.41)	1
Lenvatinib, n (%)	0 (0)	3 (0.62)	1
Sorafenib, n (%)	0 (0)	92 (18.93)	0.084
Immunotherapy, n (%)	1 (7.14)	30 (6.17)	0.597
Diabetes, n (%)	3 (21.43)	126 (25.93)	1
Hypertension, n (%)	7 (50)	297 (61.11)	0.401
Dyslipidemia, n (%)	4 (28.57)	137 (28.19)	1
AFP ≥ 100 ng/mL, n (%)	7/13 (53.85)	149/475 (31.37)	0.128

Abbreviations: MTM: macrotrabecular-massive; HCC: hepatocellular carcinoma; NASH: non-alcoholic steatohepatitis; HCV: hepatitis C virus; HBV: hepatitis B virus; AIH: autoimmune hepatitis; BCLC: Barcelona Clinic Liver Cancer; AFP: alpha-fetoprotein; RFA: radiofrequency ablation; MWA: microwave ablation; TACE: transarterial chemoembolization; SIRT: selective internal radiation therapy; LT: liver transplantation; nr: number.

**Table 2:** Univariate analysis of baseline clinical and tumor characteristics according to recurrence status.

Event, Recurrence:	Without Recurrence (*n* = 97)	With Recurrence (*n* = 120)	*p*
Sex (female), n (%)	23 (23.71)	37 (30.83)	0.244
NASH, n (%)	25 (25.77)	22 (18.33)	0.186
Cryptogen, n (%)	6 (6.19)	11 (9.17)	0.416
HCV, n (%)	37 (38.14)	45 (37.5)	0.922
HBV, n (%)	16 (16.49)	30 (25)	0.127
Alcohol, n (%)	18 (18.56)	27 (22.5)	0.476
AIH, n (%)	1 (1.03)	1 (0.83)	1
Other etiologies, n (%)	25 (25.77)	21 (17.5)	0.138
Cirrhosis, n (%)	63 (64.95)	91 (75.83)	0.079
Child–Pugh score, n (%)			0.017
A	63 (64.95)	88 (73.33)	
B	0 (0)	5 (4.17)	
No cirrhosis	34 (35.05)	27 (22.5)	
Esophageal varices, n (%)			0.003
No	84 (86.6)	90 (75)	
Yes, with bleeding	4 (4.12)	1 (0.83)	
Yes	9 (9.28)	29 (24.17)	
Ascites, n (%)	2 (2.06)	8 (6.67)	0.191
Splenomegaly, n (%)	39 (40.21)	73 (60.83)	0.003
BCLC, n (%)			0.037
0	27 (27.84)	19 (15.83)	
A	68 (70.1)	91 (75.83)	
B	2 (2.06)	6 (5)	
C	0 (0)	4 (3.33)	
D	0 (0)	0 (0)	
BCLC group (early), nr (%)	95 (97.94)	110 (91.67)	0.044
ALBI, median (IQR)	−2.67 (−3.12–−2.29)	−2.57 (−2.91–−2.19)	0.023
Portal vein invasion, n (%)	27 (27.84)	59 (70.8)	0.155
Satellite nodules, n (%)	9 (8.73)	12 (14.4)	0.925
Microvascular invasion, n (%)	35 (33.95)	54 (64.8)	0.134
Edmondson–Steiner grade, n (%)			0.371
	0 (0)	0 (0)	
I	9 (9.28)	8 (6.67)	
I/II	6 (6.19)	12 (10)	
II	43 (44.33)	40 (33.33)	
II/III	24 (24.74)	33 (27.5)	
III	13 (13.4)	25 (20.83)	
III/IV	1 (1.03)	2 (1.67)	
IV	1 (1.03)	0 (0)	
Resection, n (%)	83 (85.57)	91 (75.83)	0.074
RFA/MWA, n (%)	13 (13.4)	68 (56.67)	<0.001
TACE, n (%)	2 (2.06)	11 (9.17)	0.028
SIRT, n (%)	0 (0)	1 (0.83)	1
Lenvatinib, n (%)	0 (0)	1 (0.83)	1
Sorafenib, n (%)	5 (5.15)	22 (18.33)	0.003
Immunotherapy, n (%)	1 (1.03)	20 (16.67)	<0.001
AFP ≥ 100 ng/mL, n (%)	17 (17.89)	27 (22.88)	0.372
MTM-HCC subtype, n (%)	3 (3.09)	5 (4.17)	0.734

Abbreviations: HCC: hepatocellular carcinoma; NASH: non-alcoholic steatohepatitis; HCV: hepatitis C virus; HBV: hepatitis B virus; AIH: autoimmune hepatitis; BCLC: Barcelona Clinic Liver Cancer; ALBI: albumin-bilirubin index; AFP: alpha-fetoprotein; RFA: radiofrequency ablation; MWA: microwave ablation; TACE: transarterial chemoembolization; SIRT: selective internal radiation therapy; MTM: macrotrabecular-massive; IQR: interquartile range; nr: number.

**Table 3:** Multivariable logistic regression analysis identifying independent predictors of recurrence.

	OR Adjusted	(95% CI)	*p*
Age ≥ 65 years	0.53	(0.27–1.01)	0.056
Child–Pugh score (A/B vs. no cirrhosis)	0.77	(0.31–1.92)	0.579
Esophageal varices	0.93	(0.37–2.36)	0.885
Splenomegaly	2.76	(1.17–6.74)	0.022
BCLC group	3.33	(0.64–26.1)	0.186
MTM-HCC subtype	3.78	(0.72–21.98)	0.116

Abbreviations: OR: odds ratio; CI: confidence interval; BCLC: Barcelona Clinic Liver Cancer; MTM: macrotrabecular-massive.

**Table 4:** Multivariable Cox analysis predicting overall survival.

	HR Adjusted	(95% CI)	*p*
Age ≥ 65 years (yes vs. no)	1.27	(1.01–1.58)	0.039
BCLC group (late vs. early)	2.89	(2.2–3.79)	<0.001
PALBI	1.51	(1.22–1.87)	<0.001
CRAFITY	1.68	(1.42–1.98)	<0.001
Edmondson–Steiner grade II/III	1.35	(1.08–1.68)	0.009
Esophageal varices	1.28	(0.99–1.66)	0.063
Splenomegaly	1.35	(1.05–1.74)	0.021
RFA/MWA	1.02	(0.78–1.32)	0.91
TACE	0.69	(0.44–1.07)	0.099
Sorafenib	1.09	(0.83–1.44)	0.536
MTM	0.94	(0.49–1.81)	0.85

HR: hazard ratio; CI: confidence interval; BCLC: Barcelona Clinic Liver Cancer; PALBI: platelet-albumin-bilirubin index; CRAFITY: C-reactive protein and alpha-fetoprotein in immunotherapy score; RFA: radiofrequency ablation; MWA: microwave ablation; TACE: transarterial chemoembolization; MTM: macrotrabecular-massive.

## Data Availability

The original contributions presented in this study are included in the article. Further inquiries can be directed to the corresponding author.

## Supplementary Materials

The following supporting information can be downloaded at https://www.mdpi.com/article/10.3390/diseases14070234/s1; Supplementary Table S1—Continuous-variable comparison between MTM-HCC and non-MTM-HCC patients; Supplementary Figure S1—Recurrence-free survival according to MTM status; Supplementary Figure S2—Overall survival according to MTM status; Supplementary Figure S3—Partial dependence plot for MELD score from the random survival forest model for overall survival; Supplementary Figure S4—Partial dependence plot for BCLC stage from the random survival forest model for overall survival.

## Abbreviations

The following abbreviations are used in this manuscript:

AFP	Alpha-fetoprotein
AIH	Autoimmune hepatitis
ALBI	Albumin-bilirubin
AUROC	Area under the receiver operating characteristic curve
BCLC	Barcelona Clinic Liver Cancer
CEUS	Contrast-enhanced ultrasound
CI	Confidence interval
CRAFITY	C-reactive protein and Alpha-Fetoprotein in ImmunoTherapY
CRP	C-reactive protein
FGF19	Fibroblast growth factor 19
HBV	Hepatitis B virus
HCC	Hepatocellular carcinoma
HCV	Hepatitis C virus
HR	Hazard ratio
IQR	Interquartile range
LT	Liver transplantation
MELD	Model for End-stage Liver Disease
MTM	Macrotrabecular-massive
MTM-HCC	Macrotrabecular-massive hepatocellular carcinoma
MWA	Microwave ablation
NASH	Non-alcoholic steatohepatitis
OOB	Out-of-bag
OR	Odds ratio
OS	Overall survival
PALBI	Platelet-Albumin-Bilirubin
PD-1	Programmed cell death protein 1
PD-(L)1	Programmed death ligand 1
RFA	Radiofrequency ablation
RFS	Recurrence-free survival
RSF	Random survival forest
SIRT	Selective internal radiation therapy
TACE	Transarterial chemoembolization
TP53	Tumor protein p53
VEGFA	Vascular endothelial growth factor A
VETC	Vessels encapsulating tumor clusters
WHO	World Health Organization
