# Supplementary material for: CRAFITY and PALBI Define a Machine Learning-Supported Prognostic Framework in Hepatocellular Carcinoma—Data from an Eastern European Cohort with Low Macrotrabecular-Massive Prevalence
Source: Diseases. 2026 Jun 29;14(7):234. doi: 10.3390/diseases14070234 (PMC13408719; doi:10.3390/diseases14070234)
Supplement: Supplementary file 1 [file diseases-14-00234-s001.zip › diseases-4395632-supplementary.pdf]

CRAFITY and PALBI Define a Machine Learning-Supported Prognostic Framework in Hepatocellular Carcinoma—Data from an Eastern European Cohort with Low Macrotrabecular-Massive Prevalence

Cristiana Grapa [1,2,†], Tudor Mocan [2,3,4,†], Daniel Leucuta [5], Rares Craciun [2,6,*], Lavinia-Patricia Mocan [7], Miroslaw T. Kornek [4,8], Emil Mois [9], Nadim Al Hajjar [9], Florin Graur [9], Teodora Mocan [1,10,‡] and Zeno Sparchez [2,6,‡]

1 Department of Physiology, "Iuliu Hațieganu" University of Medicine and Pharmacy, 400012 Cluj-Napoca, Romania; grapa.cristiana.maria@elearn.umfcluj.ro (C.G.); teodora.mocan@elearn.umfcluj.ro (T.M.)
2 Gastroenterology Clinic, "Prof. Dr. O. Fodor" Regional Institute of Gastroenterology and Hepatology, 400162 Cluj-Napoca, Romania; tudor.mocan@ubbcluj.ro (T.M.); zsparchez@elearn.umfcluj.ro (Z.S.)
3 UBBmed Department, Babeș-Bolyai University, 400084 Cluj-Napoca, Romania
4 Institute of Molecular Medicine and Experimental Immunology, University Hospital of the Rheinische Friedrich-Wilhelms-University, 53127 Bonn, Germany; miroslawkornek@web.de
5 Department of Medical Informatics and Biostatistics, "Iuliu Haţieganu" University of Medicine and Pharmacy, Pasteur Street, No. 6, 400349 Cluj-Napoca, Romania; dleucuta@umfcluj.ro
6 Department of Internal Medicine, "Iuliu Hațieganu" University of Medicine and Pharmacy, 400012 Cluj-Napoca, Romania
7 Department of Histology, "Iuliu Hațieganu" University of Medicine and Pharmacy, 400349 Cluj-Napoca, Romania; trica.lavinia@umfcluj.ro
8 Department of General, Visceral and Thoracic Surgery, German Armed ForcesCentral Hospital, 56072 Koblenz, Germany
9 Department of Surgery, "Iuliu Hațieganu" University of Medicine and Pharmacy, 400162 Cluj-Napoca, Romania; drmoisemil@elearn.umfcluj.ro (E.M.); nadim.alhajjar@umfcluj.ro (N.A.H.); florin.graur@umfcluj.ro (F.G.)
10 Nanomedicine Department, Regional Institute of Gastroenterology and Hepatology, 400162 Cluj-Napoca, Romania
* Correspondence: craciun.rares.calin@elearn.umfcluj.ro
† These authors contributed equally to this work.
‡ These authors also contributed equally to this work.

Contents:

Supplementary Table S1 - Continuous-variable comparison between MTM-HCC and non-MTM-HCC patients

Supplementary Figure S1 -Recurrence-free survival according to MTM status

Supplementary Figure S2 - Overall survival according to MTM status

Supplementary Figure S3 - Partial dependence plot for MELD score from the random survival forest model for overall survival

Supplementary Figure S4 - Partial dependence plot for BCLC stage from the random survival forest model for overall survival.

Table S1. Continuous-variable comparison between MTM-HCC and non-MTM-HCC patients. Data are presented as median (interquartile range).

**MTM-HCC subtype**	**No (n=486)**	**Yes (n=14)**	**p**
Age at Diagnosis	65 (60 - 70)	68 (62.75 - 73.5)	0.258
ECOG	0 (0 - 1)	0 (0 - 0)	0.655
MELD score	9 (8 - 12)	10 (8.25 - 11.75)	0.695

ALBI	-2.53 (-2.87 - -2.07)	-2.4 (-2.85 - -2.09)	0.942
ALBI score	2 (1 - 2)	2 (1 - 2)	0.748
Fibroscan (Kpa)	17.95 (10.28 - 24.7)	12.15 (6.8 - 16.7)	0.134
BMI (kg/m2)	29 (26 - 31)	29.5 (27.25 - 31.75)	0.463
WBC (x10^3/microL)	6.9 (5.1 - 9)	6.7 (5.4 - 8.75)	1
Hb (g/dL)	13.2 (11.8 - 14.7)	12.15 (9.9 - 13.2)	***0.026***
Htc (%)	38.95 (34.8 - 43)	35.45 (31 - 39.05)	***0.038***
Platelets (x10^3/microL)	152 (104 - 214)	198 (147.5 - 251.25)	0.072
INR	1.19 (1.08 - 1.32)	1.21 (1.04 - 1.36)	0.933
Sodium (mEq/L)	139 (137 - 141)	138.5 (136.25 - 140)	0.98
Creatinin (mg/dL)	0.8 (0.67 - 0.95)	0.82 (0.67 - 1.24)	0.616
Bilirubin total (mg/dL)	0.98 (0.6 - 1.5)	0.98 (0.68 - 1.21)	0.707
AST (U/L)	55 (35 - 99.75)	48 (33.25 - 141.25)	0.941
ALT (U/L)	49 (29.25 - 89)	52.5 (27 - 124.25)	0.719
AP (U/L)	220.5 (128.25 - 319.5)	203.5 (157.5 - 341.75)	0.764
GGT (U/L)	72 (42 - 148)	88.5 (55 - 132.75)	0.377
Albumin (g/dL)	3.9 (3.4 - 4.2)	3.6 (3.45 - 4.27)	0.853
CRP (mg/dL)	1.2 (0.44 - 3.44)	1.69 (0.56 - 4.1)	0.633
AFP (ng/mL)	18.4 (4.9 - 173.85)	102.6 (3.15 - 396)	0.756
CEA (ng/mL)	2.5 (1.71 - 4.23)	2.66 (2.66 - 2.66)	0.923

Abbrevations: IQR, interquartile range; ECOG, Eastern Cooperative Oncology Group performance status; MELD, Model for End-stage Liver Disease; ALBI, Albumin-Bilirubin score; BMI, body mass index; WBC, white blood cell count; Hb, hemoglobin; Htc, hematocrit; INR, international normalized ratio; AST, aspartate aminotransferase; ALT, alanine aminotransferase; AP, alkaline phosphatase; GGT, gamma-glutamyl transferase; CRP, C-reactive protein; AFP, alpha-fetoprotein; CEA, carcinoembryonic antigen; MTM-HCC, macrotrabecular-massive hepatocellular carcinoma.

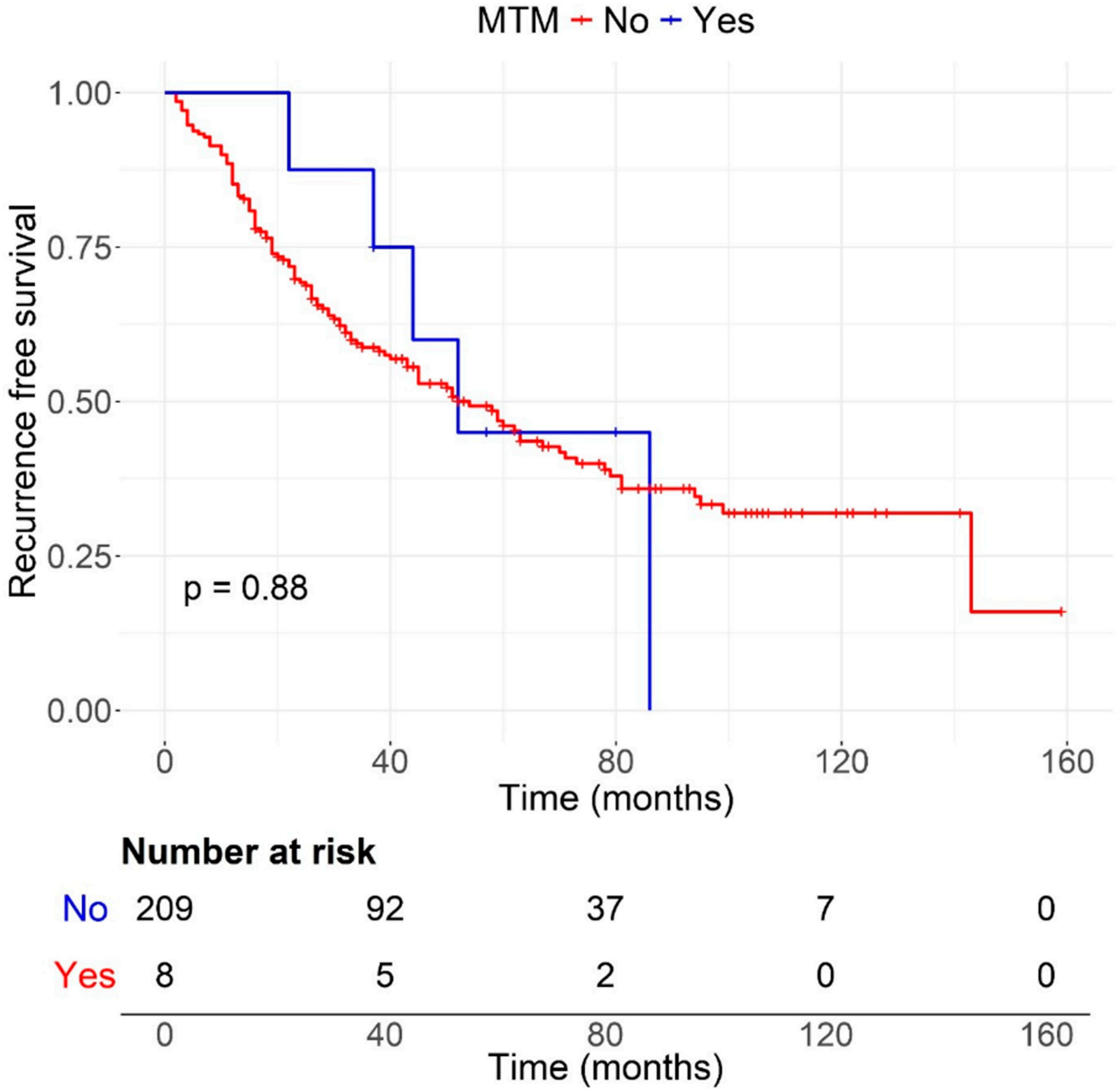

Figure S1. Recurrence free survival as a function of MTM. Recurrence-free survival was compared between patients with macrotrabecular-massive hepatocellular carcinoma (MTM-HCC) and non-MTM-HCC. Survival probabilities were estimated using the Kaplan–Meier method and compared using the log-rank test.

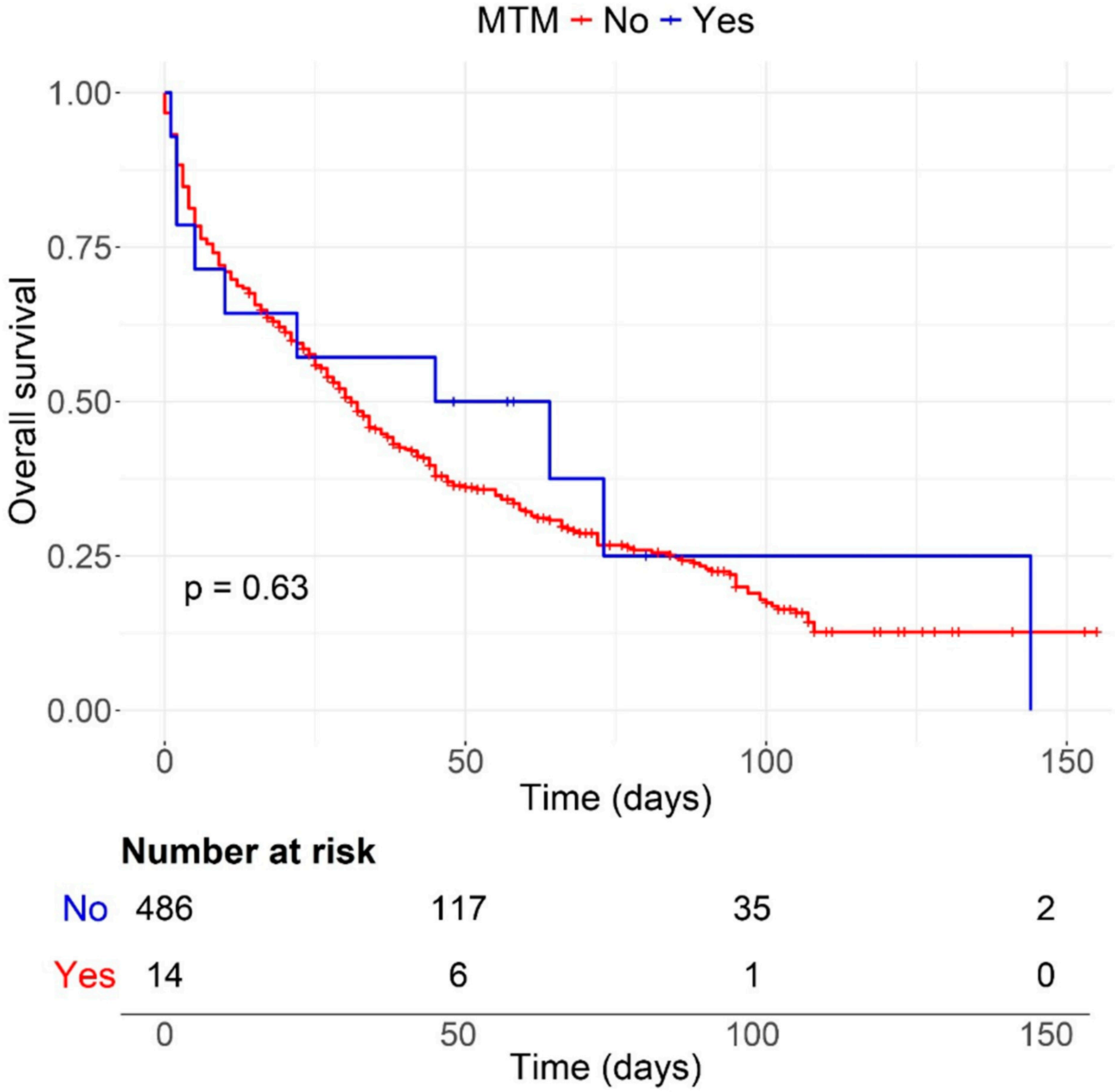

Figure S2. Overall survival as a function of MTM. Overall survival was compared between patients with macrotrabecular-massive hepatocellular carcinoma (MTM-HCC) and non-MTM-HCC. Survival probabilities were estimated using the Kaplan–Meier method and compared using the log-rank test.

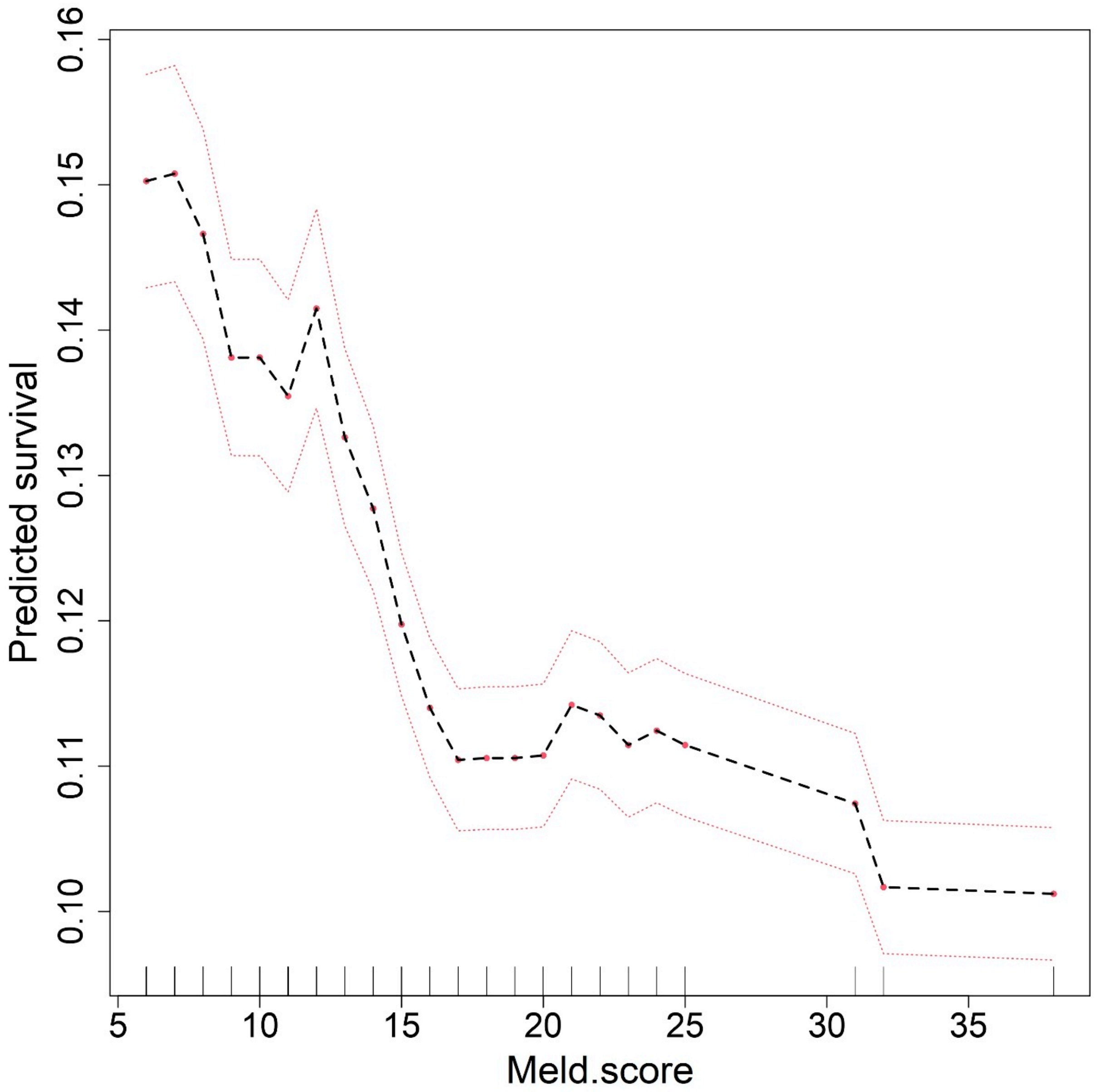

Figure S3. Partial dependence plot for Meld score. Increasing Model for End-stage Liver Disease (MELD) scores were associated with progressively lower predicted survival probabilities, with evidence of nonlinear threshold effects at higher score values. The curve represents the marginal effect of MELD on model-predicted survival while averaging over all other variables included in the model.

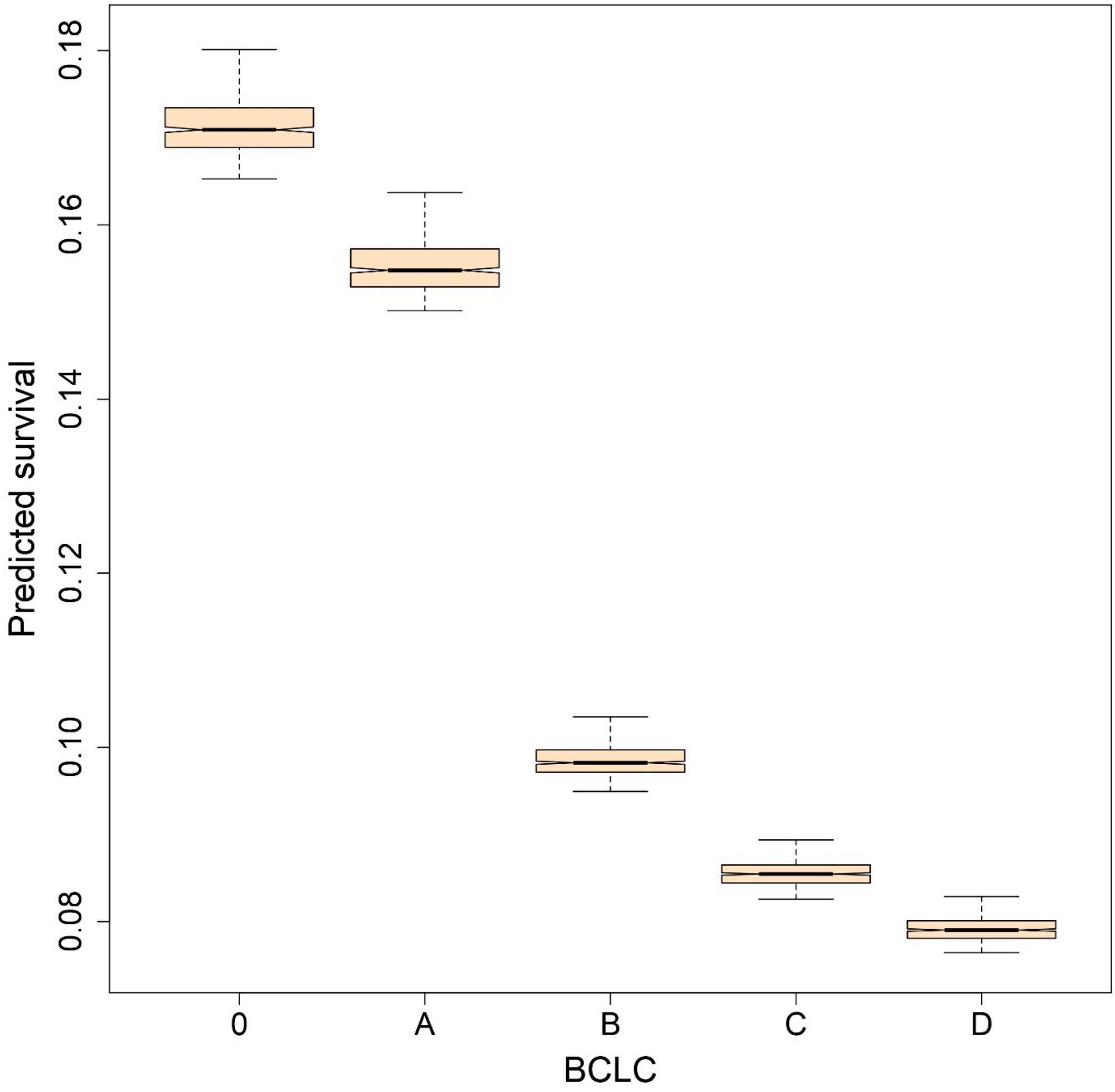

Figure S4. Partial dependence plot for BCLC. Barcelona Clinic Liver Cancer (BCLC) stage demonstrated a monotonic association with predicted survival, with progressively lower survival probabilities observed from early to advanced stages, confirming its dominant contribution to model performance within the random survival forest analysis.
